# Identifying extreme COVID-19 mortality risks in English small areas: a disease cluster approach

**DOI:** 10.1007/s00477-022-02175-5

**Published:** 2022-01-20

**Authors:** A. Adin, P. Congdon, G. Santafé, M. D. Ugarte

**Affiliations:** 1 Department of Statistics, Computer Science and Mathematics, Public University of Navarre, Pamplona, Spain; 2 Institute for Advanced Materials and Mathematics (INAMAT2), Public University of Navarre, Pamplona, Spain; 3 School of Geography, Queen Mary University of London, London, UK

**Keywords:** Disease mapping, Ecological regression, INLA, Restricted regression, Smoothing

## Abstract

The COVID-19 pandemic is having a huge impact worldwide and has highlighted the extent of health inequalities between countries but also in small areas within a country. Identifying areas with high mortality is important both of public health mitigation in COVID-19 outbreaks, and of longer term efforts to tackle social inequalities in health. In this paper we consider different statistical models and an extension of a recent method to analyze COVID-19 related mortality in English small areas during the first wave of the epidemic in the first half of 2020. We seek to identify hotspots, and where they are most geographically concentrated, taking account of observed area factors as well as spatial correlation and clustering in regression residuals, while also allowing for spatial discontinuities. Results show an excess of COVID-19 mortality cases in small areas surrounding London and in other small areas in North-East and and North-West of England. Models alleviating spatial confounding show ethnic isolation, air quality and area morbidity covariates having a significant and broadly similar impact on COVID-19 mortality, whereas nursing home location seems to be slightly less important.

## Introduction

The COVID-19 epidemic has highlighted the extent of disease inequalities between different small areas within countries, and identifying higher risk areas is an important aspect both of public health mitigation in infectious disease outbreaks, and of longer term efforts to tackle social inequalities in health. Research into spatial inequalities in COVID-19 incidence and mortality draws on a longer tradition of ecological research into health inequities. Ecological research examines the impact of area social and physical environments on population health, and seeks to establish areas with high disease risk (Roux 2016; Correa-Agudelo et al. 2021; Morenoff and Lynch 2004; Berkowitz et al. 2020). Spatial clustering in area risk factors, whether observed or unobserved, is likely to produce geographic concentrations in excess risk. For example, in a study of COVID-19 mortality in Italian municipalities the authors Ciminelli and Garcia-Mandicó (2020) find that relatively few municipalities account for a disproportionate number of deaths. An official UK study into geographic concentrations of COVID-19 mortality (Office of National Statistics (ONS) 2020a) reported that “a few areas saw COVID-19 mortality more than seven times the expected level compared with the rest of the country”. Another UK study (Kontopantelis et al. 2021) reported disproportionate concentrations of excess mortality due to COVID-19 in some regions.

Diverse methodologies have contributed to recent developments in ecological research and to assessing area health risks, including Bayesian disease mapping or BDM (Kang et al. 2016). Disease mapping uses statistical models which recognize the spatial pattern present in disease rates (e.g. geographically close areas tend to have similar disease rates) through use of random effects, and offers methods to formally identify extreme risk (Stern and Cressie 1999). One aim of such research is to smooth erratic fluctuations in risk arising from small populations and stochastic variation in disease counts. However, these procedures may sometimes produce over-smoothing, masking distinctive features in the disease risk surface, including sharp discontinuities (Duncan and Mengersen 2020). Refinements of the basic BDM models to counter this include the use of different neighborhood matrix specifications for spatial and spatio-temporal model fitting (Briz-Redón et al. 2021) or attempts to identifying clusters or individual areas exhibiting discontinuity (Knorr-Held and Rasser 2000; Anderson et al. 2014; Santafé et al. 2021). Ecological regression involving analysis of health outcomes should ideally use a relatively small area scale. Pinzari et al. (2018) mention that, to avoid attenuating impacts of area characteristics, “units with greater social homogeneity would be appropriate for studying the associations between unit characteristics and a given health indicator”. In a study of geographic obesity variations the authors Procter et al. (2008) argue that “operating at purely a global scale, say for a whole city, will ‘average out’ small areas of high prevalence such that the mean can be deemed acceptable and the pockets of problem areas are ignored, or rather, not noticed”. Geographically disaggregated models of COVID-19 outcomes have been quite widely applied (Karmakar et al. 2021; Gaudart et al. 2021; Ciminelli and Garcia-Mandicó 2020). For example, Karmakar et al. (2021) consider variations in COVID-19 incidence and mortality between US counties; this scale of analysis has the caveat that US counties vary considerably in population size, meaning some counties may contain considerable outcome heterogeneity within their boundaries. Jalilian and Mateu (2021) study variations in the daily number of new COVID-19 confirmed cases in first-level administrative division units from Spain, Italy and Germany. Gaudart et al. (2021) consider variation in COVID-19 across 96 administrative departments in France, while Ciminelli and Garcia-Mandicó (2020) consider a sample of 1161 Italian municipalities in the seven regions most severely hit by COVID-19. Several ecological regression models have been considered to estimate COVID-19 mortality risks at various geographic scales in the UK. The analysis of excess COVID-19 mortality by Kontopantelis et al. (2021) uses ten regions in England and Wales, while Travaglio et al. (2021) use data for English local authorities, averaging around 200 thousand population. The latter study found higher air pollution led to large increases in COVID-19 infectivity and mortality rates after controlling for demographic factors and health-related preconditions. Some UK analyses have been at small area level: for example, Harris (2020) considered COVID-19 mortality within the London region at the level of middle super output areas (MSOAs). MSOAs are census units averaging around 8300 population across England, with a 95th percentile population of 11,900. The study by Daras et al. (2021) was also at MSOA level, but across all of England, and found COVID-19 area vulnerability to relate to ethnic composition, poverty, prevalence of long-term health conditions, living in care homes and living in overcrowded housing. As mentioned above, risk factors such as pollution, ethnic composition and poverty have been identified as area risk factors for COVID-19 in several studies. These are likely to be spatially concentrated (for example, pollution is higher in highly urbanized areas), and so one may anticipate spatial clustering in excess risk of COVID-19 outcomes. Discontinuities may also be present, due to factors such as location of food processing plants (Food and Environment Reporting Network (FERN) 2021; Davies 2020-27-09); particular types of institution, such as prisons (Braithwaite et al. 2021); or segmented housing patterns, such as suburban social housing estates set in mainly owner occupied areas (White 2000).

In this paper we consider several classical disease mapping models and an extension of the clustering method named DBSC (Santafé et al. 2021) to an analysis of COVID-19 related mortality in English small areas during the first wave of the epidemic in the first half of 2020. We seek to identify high risk areas, and where they are most geographically concentrated, taking account of observed area factors (e.g. pre-existing illness, ethnic composition) via regression, as well as spatial correlation and clustering in regression residuals, while also allowing for spatial discontinuities. Identifying associations between the risk of mortality and several covariates, alleviating spatial confounding, i.e., avoiding collinearity between fixed and random effects, is also of interest.

The rest of the paper is structured as follows. Section 2 describes the methodology used to analyze the COVID-19 data. All the results are provided in Sect. 3. The paper ends with a discussion.

## Methods

### Choice of covariates

Choice of covariates is important in defining regression residuals which are the input to the clustering stage used later. For area covariates, we consider results from a study of COVID-19 mortality (Congdon 2021), across 6791 MSOAs (providing entire coverage of England), and covering March to June 2020 inclusive. This study found a measure of ethnic segregation to provide a better fitting model than one using simply the area percentage in ethnic groups. The following covariates have been included in the model as potential area-level risk factors: the Lieberson isolation index (ISOL) for measuring ethnic segregation; nursing home location (NH) to represent concentrations of frail elderly; a health deprivation and disability index (HDD) as a spatial measure of long term illness levels; and a measure of poor air quality (AIRQ). These variables are all continuous, and in the regression analysis they are all coded in such a way as to be “positive” risk factors, with higher scores expected to be associated with higher mortality. ISOL is a segregation measure with theoretical minimum and maximum values of 0 and 1 respectively (higher for increased segregation); the NH scores were originally percentages, increasing in line with percentages of older people in nursing homes; while AIRQ is measured as a continuous positive scale, increasing as air quality worsens. The HDD for MSOAs is obtained using HDD ranks for smaller area units, known as lower super output areas (LSOAs) and nested within MSOAs. The ranks, from 1 to 32,844, are ascending as health deprivation lessens, and are averaged within MSOAs to provide a score ranging from 35.4 to 32,835.6. These HDD scores are reversed and standardized in the regression, to provide a positive risk factor score. Table 1 provides summary descriptive statistics of these variables.

**Table 1:** Descriptive statistics of predictor variables (risk factors)

	ISOL	NH	HDD	AIRQ
Average	0.148	3.49	16,508.6	26.1
Standard deviation	0.185	3.38	8475.3	19.7
Minimum	0.005	0.00	35.4	0.4
Maximum	0.946	41.10	32,835.6	99.7

*ISOL:* Lieberson isolation index; *NH:* nursing home location; *HDD:* health deprivation and disability index; *AIRQ:* poor air quality

The COVID-19 deaths data is associated with the online article by the UK Office of National Statistics entitled “Deaths involving COVID-19 by local area and socioeconomic deprivation: deaths occurring between 1 March and 31 July 2020” (Office of National Statistics (ONS) 2020b). Data on ethnicity and nursing homes are from the UK Census, data on health deprivation are from a 2019 compendium of different types of small area deprivation (Ministry of Housing, Communities and Local Government (MHCLG) 2019), while data on air pollution are from the Access to Healthy Assets and Hazards small area indicators profile at https://www.cdrc.ac.uk/new-update-access-to-healthy-assets-and-hazards-ahah-data-resource/ (Green et al. 2018).

### Predicting relative risk: preliminary regressions

We first consider conventional spatial regression of COVID-19 mortality as a preliminar analysis and to study the best representation of the baseline spatial random effect structure.

Let $O_i$ denote observed mortality data (counts of COVID-19 related deaths) in the *i*-th MSOA during March to June 2020. The following spatial Poisson mixed model is considered

1 $$\begin{aligned} O_i | r_i&\sim {\textit{Poisson}}(E_i r_i), \quad i=1,\ldots ,n \\ \log (r_i)&= \alpha + {{\mathbf{x}}}_i^{'}{\beta } + {{\xi }_{i}} \end{aligned}$$

where $E_i$ denotes the expected number of deaths. These are computed using age specific national COVID-19 mortality rates applied to MSOA populations, so that impacts of population age structure on deaths are controlled for in the analysis; $\alpha$ is an intercept term; $r_i$ is the relative risk in area *i* (with the England wide relative risk being 1); ${{\mathbf{x}}}_i^{'}=(x_{i1}, \ldots , x_{i4})$ is the vector of standardized covariates in area *i*; ${\beta }=(\beta _1,\ldots ,\beta _4)^{'}$ is the vector of fixed effects coefficients; and $\xi _i$ is a spatial random effect. We use standardized forms of the four risk factors, putting them on a common scale so that their relative effects can be assessed (Gelman 2008). For example, we might wish to assess which risk factor is the most important influence on COVID-19 mortality.

The random effect is spatially structured to reflect possible geographic clustering in regression residuals, and a conditional autoregressive (CAR) prior is usually assumed (Besag et al. 1991; Lee 2011). In CAR priors the spatial effect for area *i* given the spatial effects in neighbouring areas is based on the average in surrounding areas (the surrounding areas may be denoted as the neighborhood or locality of area *i*); estimated risks in area *i* are smoothed towards the locality average. Different prior distributions for the spatially structured random effect $\xi$ have been proposed and there is still debate about which is the most effective at detecting risk variations (Lee 2011; Riebler et al. 2016). Some variation in area disease risk may be spatially unstructured, and this motivates models allowing for unstructured heterogeneity as well as spatial clustering in risk. Thus, the long established intrinsic CAR (iCAR) prior (Besag et al. 1991) represents pure spatial dependence. However, greater flexibility may be provided by other area priors. For instance, the Leroux CAR (LCAR) prior (Leroux et al. 1999) includes a parameter $\lambda _s$, varying between 0 and 1, to represent the proportion of risk variation that is spatially structured. This LCAR prior only includes a single random effect. By contrast, the convolution CAR prior, often termed the BYM model (Besag et al. 1991), includes a random effect for unstructured heterogeneity as well as an spatially structured random effect. Additionally, a modification of the Dean et al. (2001) model proposed by Riebler et al. (2016), hereafter called the BYM2 model, addresses both identifiability and scaling issues of the BYM model. Scaling the model is crucial to ensure that the priors for the precision parameters have the same interpretation irrespective of the spatial graph. LCAR and BYM2 also deal with spatially structured and unstructured heterogeneity, although in a different way than the BYM model does: the LCAR model through the precision matrix, and the BYM2 model uses the covariance model instead (see for example Vicente et al. 2020 for details).

As well as the standard regression estimation used in disease mapping, we also consider restricted regression (RR) models (Reich et al. 2006). RR models ensure the random effects are orthogonal to the fixed effects, and so alleviate spatial confounding (see for example, Adin et al. 2021 and references therein). Spatial confounding (meaning that the spatial random effect is collinear with the observed covariates) may attenuate or bias regression coefficients on the observed covariates, and inflate the variance of the estimates of these coefficients (Prates et al. 2019). As it can be seen in Sect. 3, similar posterior distributions of the regression coefficients to those given by the non-spatial model (GLM) are obtained when fitting RR-models, with similar relative risk estimates in relation to non-RR models as expected.

For completeness, results from a non-spatial generalized linear model (GLM) are shown also. As measures of fit, we adopt the Deviance Information Criterion (DIC) (Spiegelhalter et al. 2002), and the widely applicable information criterion (WAIC) (Watanabe 2010). These are both lower for better fitting models. Predictive fit (cross-validation outside the sample) is measured using two score statistics: Dawid–Sebastiani score, denoted DSS, and the logarithmic score, denoted LS (Czado et al. 2009). These are also lower for better fitting models. We initially consider a preliminary regression on the four area risk factors mentioned above, before investigating clustering in risks beyond that represented by the CAR random effect(s), for example due to risk discontinuities. Model fitting and inference is carried out using the very popular integrated nested Laplace approximation technique (INLA, Rue et al. 2009). All computations are made using the simplified Laplace approximation strategy of the R-INLA package (stable version 21.02.23). Regarding model hyperparameters, Normal prior distributions with mean 0 and variance equal to 1000 for fixed effects and uniform prior distributions on the positive real line for the standard deviations of the random effects have been adopted. A uniform(0, 1) prior for the spatial autocorrelation parameter $\lambda _s$ was also assumed.

We adopt commonly used, relatively diffuse, priors to summarize existing knowledge. COVID-19 is a novel infectious disease, and while there is accumulated evidence on impacts of area risk factors on respiratory diseases, there is still considerable debate and uncertainty regarding impacts of area attributes on COVID-19 mortality. Hence strongly informative priors, such as assuming that some regression coefficients are positive, are avoided. In the analysis here, the observed data has over 6000 cases (i.e., the 6791 neighbourhoods), so the likelihood will tend to dominate the influence of priors, unless these are highly informative.

There has been some effort to develop “objective” or “rule-based” priors in Bayesian analysis (Consonni et al. 2018), especially in the absence of strong prior information. For example, under the maximum entropy approach (Jaynes 2003; Schroeder 2010), suppose our background knowledge is limited to the parameter being continuous with finite mean and finite variance. Then a normal prior distribution is the maximum entropy prior, and in the analysis here we assume normal prior densities with means of zero and large finite variances to express uncertainty about the direction and variability of regression effects.

### Excess risk detection with models including cluster-level random effects

Estimation of spatial regressions may show relatively high proportions of risk variation due to unstructured heterogeneity. This component of variation may contain important information about risk patterns that is not captured by the smoothly varying spatial random effect. There are also likely to be irregularities in disease patterns such that smoothing towards the locality average (a central feature in the spatial random effect modelling) is not appropriate. For example, a deprived area with relatively poor health may be surrounded by relatively affluent areas. Hence, a novel density-based spatial clustering (DBSC) algorithm was proposed in Santafé et al. (2021) to deal with discontinuities and to smooth noisy risks in small areas. Here, we extend the proposed methodology to the context of ecological regression models as follows. In a first stage, the DBSC algorithm is used to obtain a single clustering partition ${{\mathbf{C}}}=\{C_1,\ldots ,C_k\}$ of the residuals of the non-spatial generalized linear model, that is,

2 $$\begin{aligned} {\hat{\epsilon }}_i=\log (O_i/E_i)-{{\mathbf{x}}}_i^{'}{\hat{\beta }}. \end{aligned}$$

To avoid numerical instabilities when the number of observed cases in a given small area is equal to zero, a small constant (0.0001) is added to the quotient $O_i/E_i$ in Eq. (2).

The DBSC algorithm automatically detects cluster centers in the residuals, ${\hat{\epsilon }}_i$, based on the idea that cluster centers are areas with high local density and relatively large distances to other areas with higher local density. This idea is implemented into a cluster-centroid score, $\gamma$, which is calculated for each area. This score depends on the area’s local density and the geographical distance to its closest area with a higher local density. Then, cluster centroids are automatically selected by detecting outliers in the cluster-centroid score $\gamma$. Thus, the number of clusters *k* is estimated by the algorithm and the final clustering partition is obtained by assigning the areas to their nearest cluster centroid. See Santafé et al. (2021) for more details. In addition, the DBSC algorithm has a user-given parameter, $\ell$, that defines the neighborhood order to compute the area’s local density. If $\ell =1$ is considered, the area’s local density is computed within its adjacent neighboring areas (i.e. areas that share a common border). However, greater values of $\ell$ can be used to extend the area’s neighborhood by considering $\ell$-order neighborhoods.

Then, in a second stage, the following model that includes both small area and cluster-level spatial random effects is fitted

3 $$\begin{aligned} \log (r_i) = \alpha + {{\mathbf{x}}}_i^{'}{\beta } + \xi _i + \psi _{j(i)} \end{aligned}$$

where $\psi _{j(i)}$ is the cluster-level spatial random effect and *j*(*i*) denotes that *i*th area is in cluster $C_j$. The same prior distribution is adopted for both spatial random effects $\xi _i$ and $\psi _{j(i)}$. As in Eq. (1), different CAR prior distributions can be considered (see Tables 5, 10, 11 and 12 in the Appendix for a comparison between the different CAR priors adopted in this paper). To achieve identifiability, the following sum-to-zero constrains are placed over the area and cluster random effects respectively, namely

$$\begin{aligned} \sum _{i=1}^n \xi _i = \sum _{j=1}^k \psi _{j(i)} = 0. \end{aligned}$$

Based on the evidence from the preliminary regressions, and considering that the clustering stage is computed using the residuals of a non-spatial model, restricted regression will be also applied in the second stage of the DBSC algorithm to alleviate confounding between fixed effects and the combined random effect $\xi _i + \psi _{j(i)}$, i.e, to avoid collinearity between fixed and random effects, making the fixed effect orthogonal to the random effects. The R code to run the DBSC algorithm and to fit the spatial models described in Sect. 2 is available at https://github.com/spatialstatisticsupna/DBSC_RR_article. It also includes the original data set and the code to reproduce all figures and tables of the present manuscript.

## Results

### Preliminary regressions

Table 2 shows fit measures under different specifications of the spatial effects in the preliminary regressions. A simple Poisson model without random effects (named GLM in the table) together with mixed Poisson models incorporating different areal random priors (iCAR, LCAR, BYM, and BYM2) have been fitted. To deal with spatial confounding the corresponding restricted regression (RR) models have been also fitted. A summary of posterior distributions for the fixed effects and model hyperparameters is presented in Tables 3 and 4 respectively. The posterior marginal distributions of the regression coefficients estimated for each model are plotted in the Appendix (Figs.3, 4, 5 and 6). There, it can be clearly seen how restricted regression alleviates spatial confounding with regard to the spatial regressions.

Examination of the spatial autocorrelation parameter $\lambda _s$ for the LCAR and the BYM2 models (see Table 4) suggests that the LCAR prior may overestimate the amount of spatial dependence in the random effect (the parameter takes values between 0 and 1, with 1 representing pure spatial dependence). In contrast, the BYM2 model gives a posterior mean estimate of 0.528 for its spatial autocorrelation parameter which also weights the spatially structured and spatially unstructured variability. In addition, the BYM2 model is better supported than the LCAR by the model fit criteria (see Table 2) with a significant improvement in mean deviance and better DIC, WAIC, LS, and DSS values. The BYM convolution model provides an estimate of 0.529 when computing the ratio between the marginal variance of the spatial error and the total of the marginal variances (one for spatial, one for heterogeneity), which could be interpreted as an approximation to the spatial autocorrelation parameter. However, we recommend to use the $\lambda _s$ parameter in a scaled BYM2 model. The BYM2 and the convolution model have similar fit measures (and both provide an improved fit over the LCAR), suggesting that unstructured heterogeneity is important in the overall pattern of COVID-19 mortality risk variation.

**Table 2:** Model selection criteria for models fitted with INLA

	$\bar{{D}}$	${p_D}$	DIC	WAIC	LS	DSS
GLM	44,421.6	5.3	44,426.9	44,439.9	22,219.9	34,661.2
iCAR	31,409.5	3296.4	34,705.8	34,779.1	18,546.5	17,765.2
iCAR+RR	31,409.5	3296.6	34,706.0	34,779.1	18,546.7	17,765.1
LCAR	31,355.4	3339.8	34,695.1	34,722.2	18,548.5	17,727.1
LCAR+RR	31,355.2	3340.2	34,695.4	34,722.0	18,548.6	17,726.8
BYM	30,910.7	3593.0	34,503.7	34,190.2	18,268.3	17,407.5
BYM+RR	30,910.4	3593.6	34,504.0	34,189.8	18,268.2	17,407.1
BYM2	30,911.0	3592.7	34,503.7	34,190.4	18,268.0	17,407.7
BYM2+RR	30,910.8	3593.9	34,504.6	34,190.2	18,268.1	17,407.2

GLM indicates the fit of a Generalized Linear Model (Poisson model) without random effects. iCAR, LCAR, BYM and BYM2 indicates the fit of a Poisson mixed model incorporating the intrinsic CAR, the Leroux CAR, the BYM and the BYM2 prior respectively, to the spatial random effect. The sufix RR is added to each name when restricted regression is applied

$\bar{{{D}}}$: mean deviance; ${{{p}}_{{D}}}$: effective number of parameters; *DIC:* deviance information criterion; *WAIC:* Watanabe–Akaike information criterion; *LS:* logarithmic score; *DSS:* David–Sebastiani score

The regression coefficients on all models show significant effects, namely 95% credible intervals confined to positive values, so all four postulated risk factors are relevant to explaining mortality variation. Covariate values are standardized, so comparing coefficients shows which are the most important risk factors: it seems that ethnic isolation, air quality and area morbidity have a broadly similar impact, whereas nursing home location is slightly less important. As can also be seen, similar posterior distributions to those obtained with the non-spatial generalized linear model (GLM) are obtained when fitting the restricted regression models. The Appendix plots show that the restricted regression coefficient estimates are more precise (narrower 95% credible intervals). This feature of the restricted regression option (in addition to controlling for spatial confounding) may be beneficial in establishing which observed risk factors are important for explaining mortality variation. It suggests that if restricted regression is not adopted, area risk factors that are relevant to risk variations are incorrectly assessed as not significant. Table 3 also suggests that impacts of ethnic isolation and poor air quality may be attenuated when there is no control for spatial confounding. High-resolution maps with the posterior median estimates of relative risks and posterior exceedence probabilities $Pr(r_i>1 | {{\mathbf{O}}})$ obtained with the BYM2 model are available at https://emi-sstcdapp.unavarra.es/England_MSOA/.

**Table 3:** Posterior mean, posterior standard deviation, and 95% credible intervals of the regression coefficients for GLM, iCAR, LCAR, BYM and BYM2 models (and the corresponding RR versions) fitted with INLA

INLA models	Mean	SD	${q_{0.025}}$	Median	${q_{0.975}}$
*Lieberson index (ISOL)*					
GLM	0.162	0.005	0.151	0.162	0.173
iCAR	0.102	0.015	0.072	0.102	0.132
LCAR	0.105	0.015	0.075	0.105	0.135
BYM	0.113	0.013	0.087	0.113	0.139
BYM2	0.113	0.013	0.087	0.113	0.139
iCAR+RR	0.153	0.006	0.142	0.153	0.164
LCAR+RR	0.153	0.006	0.142	0.153	0.164
BYM+RR	0.153	0.006	0.142	0.153	0.164
BYM2+RR	0.153	0.006	0.142	0.153	0.164
*Nursing homes (NH)*					
GLM	0.087	0.004	0.079	0.087	0.095
iCAR	0.115	0.007	0.101	0.115	0.129
LCAR	0.114	0.007	0.100	0.114	0.128
BYM	0.107	0.007	0.092	0.107	0.121
BYM2	0.107	0.007	0.092	0.107	0.121
iCAR+RR	0.100	0.004	0.092	0.100	0.108
LCAR+RR	0.100	0.004	0.092	0.100	0.108
BYM+RR	0.101	0.004	0.092	0.101	0.109
BYM2+RR	0.101	0.004	0.092	0.101	0.109
*Health deprivation and disability (HDD)*					
GLM	0.163	0.005	0.154	0.163	0.172
iCAR	0.195	0.013	0.170	0.195	0.220
LCAR	0.188	0.012	0.164	0.188	0.213
BYM	0.192	0.012	0.169	0.192	0.216
BYM2	0.192	0.012	0.169	0.192	0.216
iCAR+RR	0.167	0.005	0.158	0.167	0.176
LCAR+RR	0.167	0.005	0.158	0.167	0.176
BYM+RR	0.166	0.005	0.157	0.166	0.175
BYM2+RR	0.166	0.005	0.157	0.166	0.175
*Air quality (AIRQ)*					
GLM	0.170	0.006	0.158	0.170	0.181
iCAR	0.071	0.038	−0.003	0.071	0.145
LCAR	0.121	0.036	0.049	0.121	0.189
BYM	0.082	0.027	0.028	0.082	0.135
BYM2	0.082	0.027	0.028	0.082	0.135
iCAR+RR	0.145	0.006	0.133	0.145	0.157
LCAR+RR	0.145	0.006	0.133	0.145	0.157
BYM+RR	0.147	0.006	0.135	0.147	0.159
BYM2+RR	0.147	0.006	0.135	0.147	0.159

**Table 4:** Posterior mean, posterior standard deviation, and 95% credible intervals of the model hyperparameters for GLM, iCAR, LCAR, BYM and BYM2 models (and the corresponding restricted regression (RR) versions) fitted with INLA

INLA models		Mean	SD	${q_{0.025}}$	Median	${q_{0.975}}$
iCAR and iCAR+RR models	$\tau _s$	1.338	0.046	1.250	1.336	1.433
LCAR and LCAR+RR models	$\tau _s$	1.331	0.046	1.242	1.330	1.425
	$\lambda _s$	0.936	0.039	0.839	0.948	0.981
BYM and BYM+RR models	$\tau _u$	7.706	0.435	6.925	7.677	8.633
	$\tau _v$	4.417	0.398	3.688	4.398	5.252
BYM2 and BYM2+RR models	$\tau _s$	3.658	0.139	3.393	3.655	3.938
	$\lambda _s$	0.528	0.032	0.464	0.528	0.591

### Risk clustering

The model selection criteria of the preliminary regressions (see Table 2) support the BYM and BYM2 options (involving an unstructured heterogeneity random effect, as well as a spatial effect that pools towards the locality average). The DBSC clustering aims to better elucidate the sources of this variability and also assesses when the principle of locality smoothing may need to be modified. Having identified clusters using the regression residuals of the simple Poisson model described in Eq. (2), we then apply an extended spatial regression model including cluster random effects $\psi _{j(i)}$ [see Eq. (3)]. Finally, we also carry out restricted regression to alleviate spatial confounding. The results obtained for different values of the $\ell$ parameter when fitting the BYM2+C models are shown in Table 5. In general, considerably improved model fit criteria are obtained as compared to the preliminary BYM2 model which do not take account of risk clustering (compare Tables 2 and 5). The option $\ell =1$ is clearly preferred. The corresponding results when fitting iCAR+C, LCAR+C and BYM2+C models are shown in Tables 10, 11 and 12 of the Appendix, respectively.

**Table 5:** Model selection criteria for BYM2+C models fitted with INLA considering different neighborhood orders (parameter $\ell$)

BYM2+C	$\bar{{D}}$	${p_D}$	DIC	WAIC	LS	DSS
${\ell =1}$	30,548.9	2417.4	32,966.3	32,879.5	16,970.6	17,222.3
${\ell =2}$	30,665.7	2655.1	33,320.8	33,204.1	17,263.6	17,282.1
${\ell =3}$	30,699.9	2724.8	33,424.7	33,297.4	17,377.9	17,304.5
${\ell =4}$	30,733.9	2713.5	33,447.4	33,328.4	17,374.8	17,329.1
${\ell =5}$	30,710.8	2829.9	33,540.7	33,363.9	17,505.2	17,296.1
${\ell =6}$	30,750.0	2790.7	33,540.7	33,393.1	17,491.0	17,338.1
${\ell =7}$	30,716.8	2810.1	33,526.9	33,379.3	17,457.3	17,302.4
${\ell =8}$	30,769.0	2912.4	33,681.3	33,516.7	17,636.6	17,332.4

**Fig. 1:**
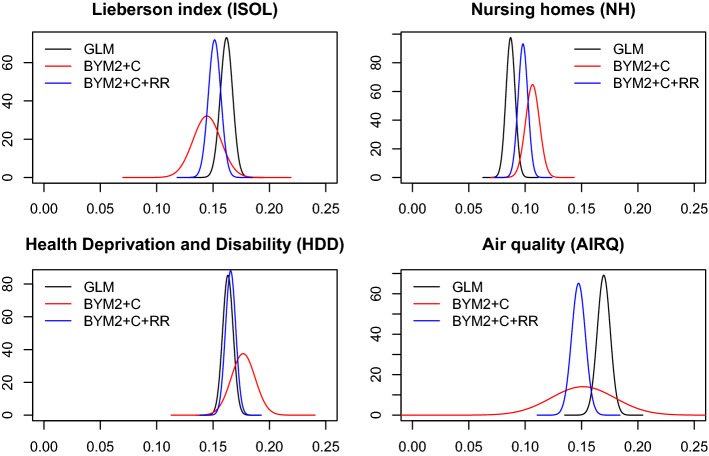
Posterior marginal distributions of the regression coefficients (BYM2+C model)

Posterior marginal distributions of the regression coefficients under the confounded and restricted regression options are shown in Table 6 and Fig. 1. As before, it can be seen that restricted regression produces more precise estimates of the regression coefficients, with ethnic isolation, area morbidity, and poor air quality again figuring as the most important observed predictors of risk variability.

**Table 6:** Posterior mean, posterior standard deviation, and 95% credible intervals of the regression coefficients for GLM, BYM2+C and BYM2+C+RR models ($\ell =1$) fitted with INLA

INLA models	Mean	SD	${q_{0.025}}$	Median	${q_{0.975}}$
*Lieberson index (ISOL)*					
GLM	0.162	0.005	0.151	0.162	0.173
BYM2+C	0.145	0.012	0.120	0.154	0.169
BYM2+C+RR	0.151	0.006	0.140	0.151	0.162
*Nursing homes (NH)*					
GLM	0.087	0.004	0.079	0.087	0.095
BYM2+C	0.107	0.006	0.094	0.107	0.119
BYM2+C+RR	0.098	0.004	0.090	0.098	0.106
*Health deprivation and disability (HDD)*					
GLM	0.163	0.005	0.154	0.163	0.172
BYM2+C	0.177	0.011	0.156	0.177	0.198
BYM2+C+RR	0.166	0.005	0.157	0.166	0.174
*Air quality (AIRQ)*					
GLM	0.170	0.006	0.158	0.170	0.181
BYM2+C	0.151	0.028	0.096	0.151	0.207
BYM2+C+RR	0.147	0.006	0.135	0.147	0.159

### Risk classifications

The geographic distribution of extreme relative risk is of importance for prioritizing areas for intervention and countering excess morbidity in future epidemics. To this end we use an eight-fold categorization of MSOAs according to both their urban-rural location (Office of National Statistics (ONS) 2013) and region of location (nine regions). We define extreme relative risk in the *i*-th MSOA using high posterior probabilities that $r_i$ exceeds 1.5, namely the exceedence probabilities $Pr(r_i> 1.5 | {{\mathbf{O}}}) > 0.9$. In words, there is a high probability that the excess of risk in the *i*-th MSOA will be at least 50% more when compared with the global risk in England. We also consider overlapping relative risk: where risk is elevated both in an MSOA itself, and also in surrounding (adjacent) MSOAs. We define this as occurring when both relevant probabilities are high, namely when $Pr(r_i> 1 | {{\mathbf{O}}}) > 0.9$, and when $Pr(R_i> 1 | {{\mathbf{O}}}) > 0.9$, where $R_i$ is the average relative risk in surrounding MSOAs (the locality of area i).

In what follows, we compare the results obtained with the conventional BYM2 model given in Eq. (1) (without a cluster random effect), with the BYM2+C based regression model given in Eq. (3) (which includes a cluster random effect). Note that same results are obtained when comparing the corresponding restricted regression models as expected.

Table 7 shows the number of MSOAs with $Pr(r_i> 1.5 | {{\mathbf{O}}}) > 0.9$ according to urban-rural category under both BYM2 and BYM2+C models. It can be seen that the latter produces a higher number of MSOAs with extremely high risk, especially in highly urbanized settings, namely 518 as against 396. The cluster regression also provides a classification of extreme risk that includes more deaths (11,086 out of a total of 49,232, or 22.5%) as against the conventional regression.

Table 8 classifies MSOAs by region. Here extremely high risk is concentrated in London, and to a lesser extent the two most Northerly regions. This feature is more clearly apparent under the clustering regression, especially in the North West region. The clustering regression also identifies more overlapping risk, again in Northern regions. High (but not necessarily extremely high) risk can be assessed on the basis of probabilities ${\textit{Pr}}(r_i> 1 | {{\mathbf{O}}}) > 0.9$. We may also consider extremely low risk areas, with relative risk below $1/1.5= 0.667$, on the basis of the probabilities ${\textit{Pr}}(r_i < 1/1.5 | {{\mathbf{O}}}) >0.9$, and low relative risk areas with ${\textit{Pr}}(r_i < 1 | {{\mathbf{O}}}) > 0.9$.

**Table 7:** Total MSOAs classified as extreme relative risk by Urban–Rural category: BYM2 vs. BYM2+C models

Urban–Rural category	Observed SMR	Number of areas with high probability ${r_i > 1.5}$ (BYM2)	Number of areas with high probability ${r_i > 1.5}$ (BYM2+C)	Deaths in areas with high probability ${r_i > 1.5}$ (BYM2)	Deaths in areas with high probability ${r_i > 1.5}$ (BYM2+C)	Total areas with high probability of overlapping risk (BYM2)	Total areas with high probability of overlapping risk (BYM2+C)
Urban: major conurbation	1.43	396	518	6471	7651	437	478
Urban: minor conurbation	1.16	14	22	296	399	1	2
Urban: city & town	0.89	115	155	2395	2838	31	51
Urban: city/Town in sparse setting	0.47	0	0	0	0	0	0
Rural: town & fringe	0.70	5	7	118	130	1	2
Rural: town & fringe in sparse setting	0.51	0	0	0	0	0	0
Rural: village & dispersed	0.59	2	3	49	68	0	1
Rural: village & dispersed in sparse setting	0.37	0	0	0	0	0	0
All MSOAs	1.00	532	705	9329	11,086	470	534

*BYM2:* conventional regression; *BYM2+C:* regression adjusted for discontinuity clustering

**Table 8:** Total MSOAs classified as extreme relative risk by English region: BYM2 vs BYM2+C models

Region	Observed SMR	Number of areas with high probability ${r_i > 1.5}$ (BYM2)	Number of areas with high probability ${r_i > 1.5}$ (BYM2+C)	Deaths in areas with high probability ${r_i > 1.5}$ (BYM2)	Deaths in areas with high probability ${r_i > 1.5}$ (BYM2+C)	Total areas with high probability of overlapping risk (BYM2)	Total areas with high probability of overlapping risk (BYM2+C)
North East	1.16	42	48	909	954	11	18
North West	1.24	89	132	1616	2118	50	76
Yorkshire–Humberside	1.01	50	67	904	1103	16	23
West Midlands	1.13	69	86	1138	1303	68	80
East Midlands	0.91	21	29	407	472	10	18
East	0.86	23	33	456	597	13	13
South East	0.84	28	40	590	717	1	1
London	1.58	204	262	3154	3637	301	304
South West	0.49	6	8	155	185	0	1
All Areas	1.00	532	705	9329	11,086	470	534

*BYM2:* conventional regression; *BYM2+C:* regression adjusted for discontinuity clustering

Table 9 shows that the conventional model classifies a much higher proportion of areas as having intermediate risk, with lower numbers of extreme high or low risk. Discrepant classifications of risk can be defined based on comparing ${\textit{Pr}}(r_i > 1.5 | {{\mathbf{O}}})$ between the cluster-adjusted and conventional models. A discrepant high risk classification is defined when ${\textit{Pr}}(r_i> 1.5 | {{\mathbf{O}}}) > 0.9$ under the BYM2+C model, but ${\textit{Pr}}(r_i > 1.5 | {{\mathbf{O}}}) < 0.8$ under the BYM2 model. There are 93 such areas, in which total deaths are 952, and total expected are 501.8, giving a point estimate of 1.90 for the standard mortality ratio, so meriting the classification as high risk. A discrepant low risk classification is defined when ${\textit{Pr}}(r_i < 0.667 | {{\mathbf{O}}}) > 0.9$ under the BYM2+C model, but ${\textit{Pr}}(r_i< 0.667 | {{\mathbf{O}}}) < 0.8$ under the BYM2 model. There are 176 such areas, with a total of 340 deaths against 683.5 expected, giving a point estimate of 0.5 for the standardized mortality ratio in these areas, and so clearly low risk areas.

**Table 9:** Relative risk categories by model: BYM2 vs BYM2+C

	BYM2 Model	% All MSOAs	BYM2+C Model	% All MSOAs
Extreme high relative risk, ${\text {Pr}}(r_i>1.5 \mid \text {O})>0.9$	532	7.8	705	10.4
Elevated (excl extremely high) relative risk, $\text {Pr}(r_i>1\mid \text {O})>0.9$	1055	15.5	1186	17.5
Intermediate relative risk, $0.9>\text {Pr}(r_i>1 \mid \mathrm {O})>0.1$	3166	46.6	2408	35.5
Low relative risk (excl extremely low), $\text {Pr}(r_i<1 \mid \mathrm {O})>0.9$	1242	18.3	1386	20.4
Extreme low relative risk, ${\text {Pr}(r_i<0.67 \mid 0)>0.9}$	796	11.7	1106	16.3
All categories	6791	100.0	6791	100.0

**Fig. 2:**
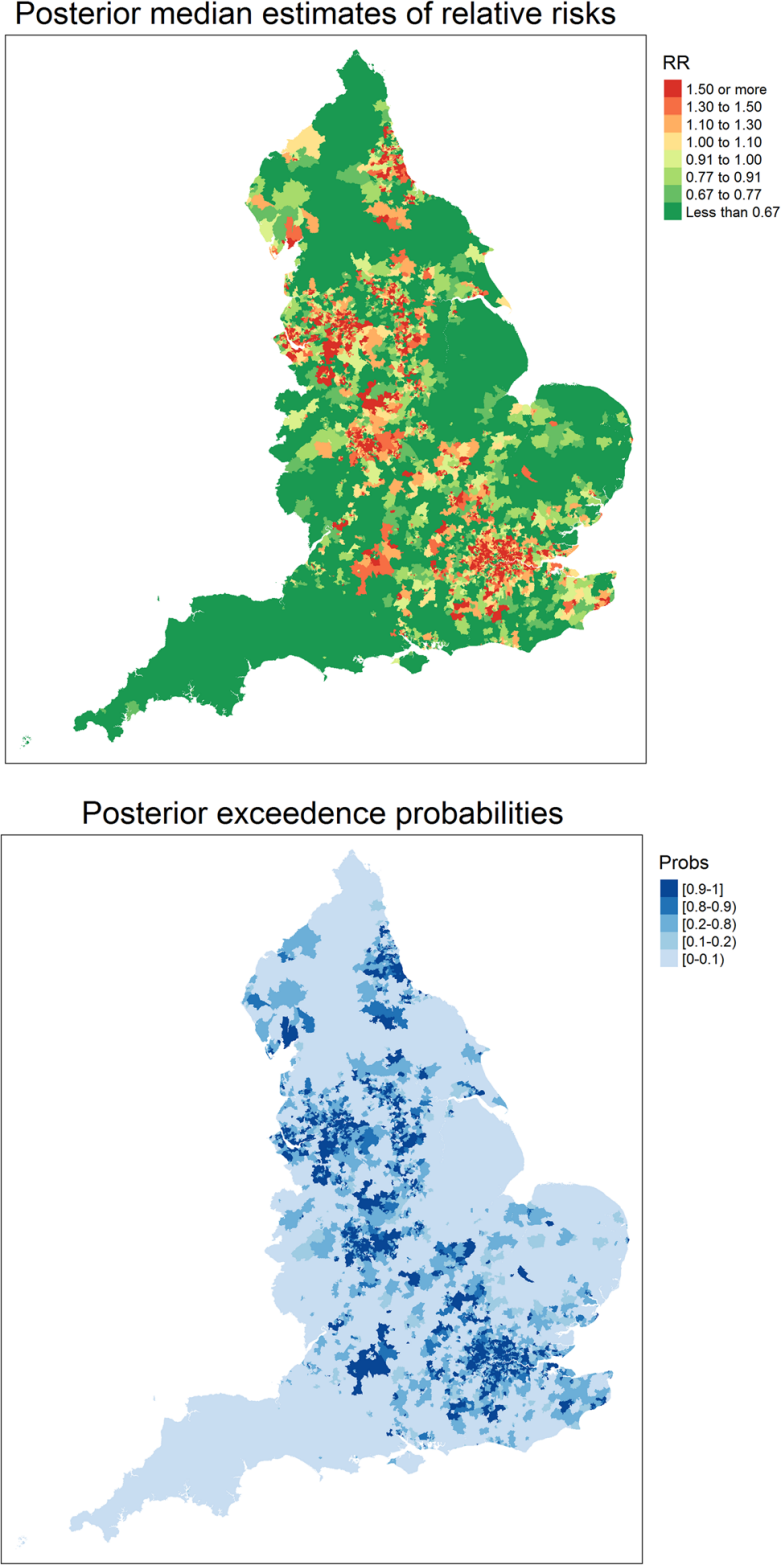
Posterior median estimates of relative risks (top) and posterior exceedence probabilities $Pr (r_i > 1 | {{\mathbf{O}}})$ (bottom) obtained with the $\ell =1$ BYM2+C model

**Fig. 3:**
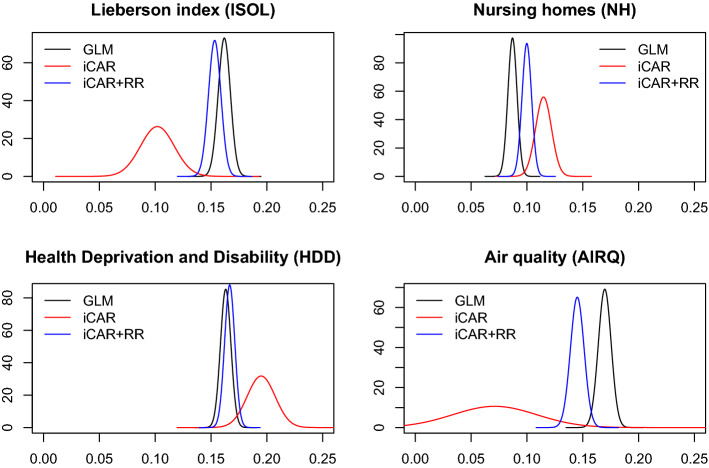
Posterior marginal distributions of the regression coefficients (iCAR model)

**Fig. 4:**
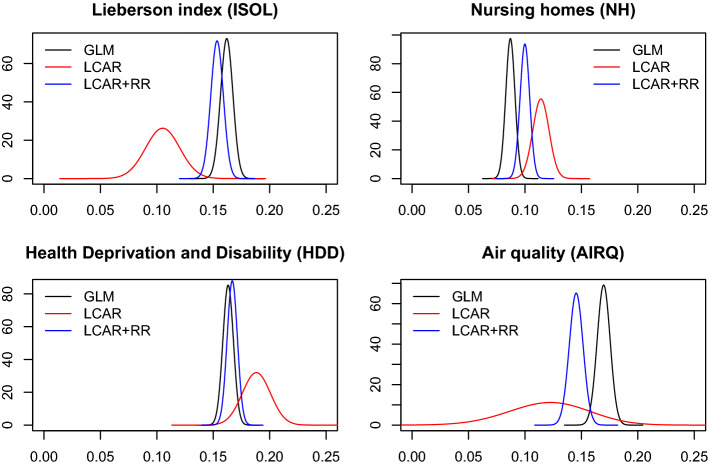
Posterior marginal distributions of the regression coefficients (LCAR model)

**Fig. 5:**
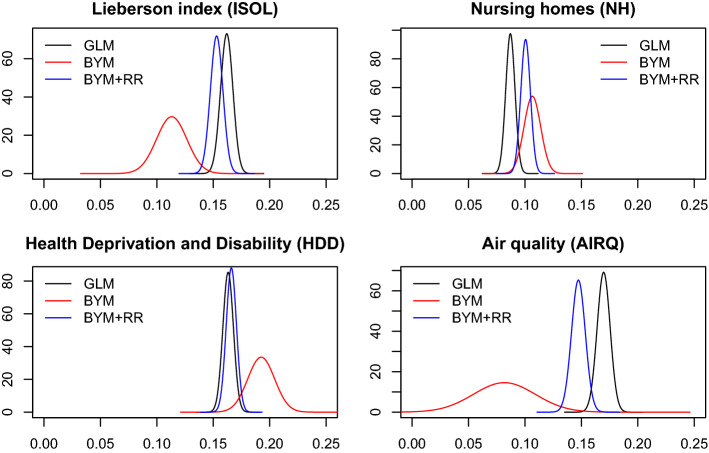
Posterior marginal distributions of the regression coefficients (BYM model)

**Fig. 6:**
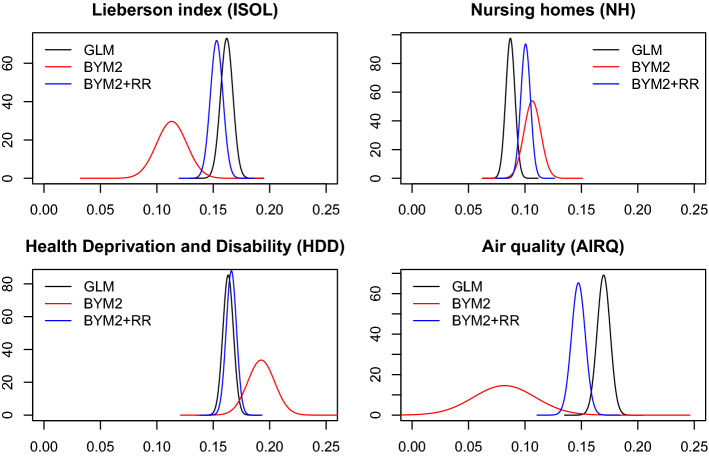
Posterior marginal distributions of the regression coefficients (BYM2 model)

Maps with the posterior median estimates of relative risks and posterior exceedence probabilities ${\textit{Pr}} (r_i > 1 | {{\mathbf{O}}})$ obtained with the BYM2+C model ($\ell =1$) are plotted at Fig. 2. High-resolution version of these maps are also available at https://emi-sstcdapp.unavarra.es/England_MSOA/.

## Discussion

Delineation of high risk areas is a primary aim in disease mapping. A disease mapping model that underpredicts cases or deaths in high risk areas may lead to resourcing decisions that do not match health need. It may also be relevant to consider distinctively low risk areas, not so much on resourcing grounds, but because the location of low risk is important in assessing which environments are favorable from the viewpoint of reducing health risk. The preceding section has compared risk classifications under a conventional disease mapping model (without a clustering term) and a model including a cluster random effect to account for discrepancies in the conventional model, which can be called a clustering adjusted model. It can be seen from Table 7 that the latter produces a higher number of MSOAs with extreme risk, especially in highly urbanized settings. The clustering adjusted regression model (and hence its classifications of risk) is considerably better supported by fit measures than the conventional model. This implies, inter alia, that the conventional model is understating extreme relative risk in urban areas. We may also consider areas with low risk, as defined by high probabilities that the relative risk is under 1, or decisively under 1, namely below 1/1.5.

Table 9 shows that the conventional disease mapping model tends to classify a noticeably higher proportion of areas (47%) as having intermediate risk, as compared to the clustering adjusted model (36%), and to understate the numbers of definitively high risk and definitively low risk areas. This suggests over-smoothing under the conventional model. If the classification produced by conventional disease mapping was used as a basis for resource allocation, then it would tend to disadvantage areas with the highest need. An examination of areas with discrepant risk classifications shows that the conventional disease mapping model provides estimated probabilities ${\textit{Pr}}(r_i >1.5| {{\mathbf{O}}}) < 0.8$ for 93 areas, despite such areas having an SMR of 1.9. Similarly the conventional disease mapping model provides estimated probabilities ${\textit{Pr}}(r_i< 0.667| {{\mathbf{O}}}) < 0.8$ for 176 areas, despite such areas having an SMR of 0.5. Examination of the discrepant risk areas suggest that the clustering adjusted model corrects for misclassification, which may occur when an area has relatively high (low) mortality as compared to its locality of surrounding areas. An unadjusted spatial smoothing mechanism (which pools to the locality average) may mean that an above average mortality area may have a relative risk estimate of below 1 if its locality has comparatively lower mortality. The DBSC clustering approach will tend to allocate such an area to a high mortality cluster to compensate for the spatial smoothing effect. This is not to discount the utility of spatial smoothing in disease mapping, but to suggest that this smoothing principle may need to be modified when there are risk discontinuities. The clustering model will also tend to adjust when an area is classified as relatively low risk on the basis of observed area risk factors (e.g. when the four predictors used to predict COVID-19 mortality are all below average), whereas other indications are of high mortality. This could be a high ratio of $O_i$ to $E_i$ in both the area itself (especially when $E_i$ is relatively high, say above 5), and also in its locality of surrounding areas.

As suggested by one reviewer, a small simulation study was conducted to compare classical models with the methodology proposed in this paper in terms of detecting extreme risks. Even simulating using the risks obtained under a BYM2 model, results suggests that our clustering adjusted regression model proposal performs better than usual CAR models when the objective is to correctly identify extreme risk areas. Specifically, higher values of true positive rates and true negative rates were obtained when considering models that also includes a cluster-level spatially structured random effect. Although a slight increase is observed in terms of false negative/positive rates, these values never overcame 2% when defining high/low risk areas as those with high posterior probabilities that risks exceed 1.5 or are below 0.67 (that is, 1/1.5), respectively.

The other methodological feature of the analysis of this paper is the benefit of comparing restricted spatial regression, which controls for spatial confounding (i.e., avoids the collinearity between fixed and random effects), with conventional disease mapping. The four area risk factors used to predict COVID-19 mortality have more precisely identified effects under restricted regression attributing all the competing explanatory effect to the covariates and considering random effects as smoothing devices. In some situations, where an area risk factor has a less clear cut effect, the gain in precision may mean the difference between deciding whether a regression effect is significant or not.

The main substantive conclusions to emerge from the analysis of this paper are a pronounced metropolitan vs rural delineation of risk, which much outweighs any North-South divide. In fact, the main difference is between London and other regions. This stands opposite to longer term health contrasts between Northern and Southern England (Buchan et al. 2017). As to establishing such contrasts, a spatial regression model incorporating a clustering stage to identify risk continuities provides a risk classification that provides a clear advantage on the basis of a range of fit measures. The conventional spatial regression, here provided by the BYM2 model of Riebler et al. (2016), tends to classify a much higher proportion of areas as having intermediate risk, and is subject to apparent misclassification of some areas. The latter is exemplified by subsets of areas with clearly elevated (or depressed) risk based on standard mortality ratios, but not classified as extreme risk. Some limitations of the analysis here may be mentioned. No risk classifications are perfect, and are subject to stochastic uncertainty. Furthermore risk classifications of areas are of population aggregates, and more localized analysis may be needed to establish which sub-areas of high risk MSOAs show most adverse risk.

## Appendix

See Figs. 3, 4, 5, 6 and Tables 10, 11, 12.

**Table 10:** Model selection criteria for iCAR+C models fitted with INLA considering different neighborhood orders (parameter $\ell$)

iCAR+C	$\bar{{D}}$	${p_D}$	DIC	WAIC	LS	DSS
${\ell =1}$	30,607.7	2389.5	32,997.2	32,946.9	17,020.1	17,270.1
${\ell =2}$	30,804.1	2554.4	33,358.5	33,331.4	17,348.2	17,403.3
${\ell =3}$	30,804.0	2633.3	33,437.3	33,381.8	17,426.2	17,397.7
${\ell =4}$	30,879.9	2581.1	33,461.0	33,434.7	17,421.8	17,467.5
${\ell =5}$	30,906.3	2642.2	33,548.5	33,514.2	17,593.0	17,485.3
${\ell =6}$	30,920.2	2653.5	33,573.8	33,532.2	17,595.8	17,501.4
${\ell =7}$	30,950.2	2624.0	33,574.2	33,567.5	17,524.0	17,523.6
${\ell =8}$	31,037.7	2695.9	33,733.6	33,740.6	17,711.5	17,583.2

**Table 11:** Model selection criteria for LCAR+C models fitted with INLA considering different neighborhood orders (parameter $\ell$)

LCAR+C	$\bar{{D}}$	${p_D}$	DIC	WAIC	LS	DSS
${\ell =1}$	30,581.4	2379.2	32,960.6	32,895.4	16,984.6	17,259.3
${\ell =2}$	30,836.0	2492.9	33,328.9	33,318.0	17,292.3	17,462.3
${\ell =3}$	30,748.2	2672.4	33,420.6	33,323.3	17,438.6	17,357.1
${\ell =4}$	30,846.7	2607.1	33,453.7	33,400.6	17,427.1	17,445.4
${\ell =5}$	30,900.7	2631.2	33,531.9	33,484.0	17,556.8	17,496.2
${\ell =6}$	30,900.6	2659.9	33,560.5	33,497.3	17,539.5	17,495.2
${\ell =7}$	30,911.2	2641.9	33,553.1	33,521.1	17,525.4	17,499.7
${\ell =8}$	30,968.7	2737.1	33,705.8	33,664.6	17,696.7	17,529.1

**Table 12:** Model selection criteria for BYM+C models fitted with INLA considering different neighborhood orders (parameter $\ell$)

BYM+C	$\bar{{D}}$	${p_D}$	DIC	WAIC	LS	DSS
${\ell =1}$	30,556.7	2395.7	32,952.4	32,876.8	16,966.0	17,233.4
${\ell =2}$	30,659.5	2622.9	33,282.4	33,170.8	17,216.5	17,277.9
${\ell =3}$	30,696.5	2711.5	33,408.0	33,280.7	17,344.9	17,301.4
${\ell =4}$	30,754.5	2679.0	33,433.5	33,333.0	17,359.5	17,352.2
${\ell =5}$	30,746.5	2760.0	33,506.5	33,368.3	17,444.5	17,332.2
${\ell =6}$	30,720.3	2787.9	33,508.3	33,349.0	17,425.2	17,308.7
${\ell =7}$	30,675.6	2812.0	33,487.6	33,321.4	17,462.7	17,264.4
${\ell =8}$	30,751.3	2882.7	33,634.1	33,473.0	17,561.6	17,313.9
